# Structural and Functional Characterization of *Legionella* *pneumophila* Effector MavL

**DOI:** 10.3390/biom11121802

**Published:** 2021-11-30

**Authors:** Kevin Voth, Shivani Pasricha, Ivy Yeuk Wah Chung, Rachelia R. Wibawa, Engku Nuraishah Huda E. Zainudin, Elizabeth L. Hartland, Miroslaw Cygler

**Affiliations:** 1 Department of Biochemistry, Microbiology & Immunology, University of Saskatchewan, 107 Wiggins Road, Saskatoon, SK S7N 5E5, Canada; kevinvoth@gmail.com (K.V.); ivy.chung@usask.ca (I.Y.W.C.); 2 Centre for Innate Immunity and Infectious Diseases, Hudson Institute of Medical Research, Clayton 3168, Australia; shivani.pasricha@unimelb.edu.au (S.P.); raissa.wibawa@hudson.org.au (R.R.W.); 3 Department of Microbiology and Immunology, University of Melbourne at the Peter Doherty Institute for Infection and Immunity, Melbourne 3000, Australia; engkunuraishah@iium.edu.my; 4 Department of Molecular and Translational Science, Monash University, Clayton 3168, Australia

**Keywords:** *Legionella* effector, crystal structure, ADP-ribosyltransferase fold, protein-protein interactions, cellular localization

## Abstract

*Legionella pneumophila* is a Gram-negative intracellular pathogen that causes Legionnaires’ disease in elderly or immunocompromised individuals. This bacterium relies on the Dot/Icm (Defective in organelle trafficking/Intracellular multiplication) Type IV Secretion System (T4SS) and a large (>330) set of effector proteins to colonize the host cell. The structural variability of these effectors allows them to disrupt many host processes. Herein, we report the crystal structure of MavL to 2.65 Å resolution. MavL adopts an ADP-ribosyltransferase (ART) fold and contains the distinctive ligand-binding cleft of ART proteins. Indeed, MavL binds ADP-ribose with Kd of 13 µM. Structural overlay of MavL with poly-(ADP-ribose) glycohydrolases (PARGs) revealed a pair of aspartate residues in MavL that align with the catalytic glutamates in PARGs. MavL also aligns with ADP-ribose “reader” proteins (proteins that recognize ADP-ribose). Since no glycohydrolase activity was observed when incubated in the presence of ADP-ribosylated PARP1, MavL may play a role as a signaling protein that binds ADP-ribose. An interaction between MavL and the mammalian ubiquitin-conjugating enzyme UBE2Q1 was revealed by yeast two-hybrid and co-immunoprecipitation experiments. This work provides structural and molecular insights to guide biochemical studies aimed at elucidating the function of MavL. Our findings support the notion that ubiquitination and ADP-ribosylation are global modifications exploited by *L. pneumophila*.

## 1. Introduction

*Legionella pneumophila* is the causative agent of a life-threatening pneumonia called Legionnaires’ disease. By infecting a broad range of protozoa in the environment, *L. pneumophila* has acquired distinct but overlapping mechanisms of virulence that rely on over 300 secreted effector proteins [1]. Arriving at a functional understanding of each of these effectors is hindered by the redundancy of their actions in the host cell [2], but is essential to provide insights into how *L. pneumophila* usurps host defenses. X-ray crystallography has already expanded our knowledge of *L. pneumophila* pathogenesis, by revealing many effectors that harbor eukaryotic-like folds and interfere with the host using molecular mimicry [3]. Structural data are especially useful in cases where the effector in question is functionally redundant, and knockout *L. pneumophila* mutants do not give rise to a phenotype.

MavL (Lpg2526) was initially identified in a study of novel type IV secretion system (T4BSS) substrates [4]. Briefly, a protein known to pass through the T4BSS (SidC) was truncated by 100 residues at the C-terminus to render it translocation deficient (SidCΔ100). Fusing the C-terminal residues of putative effectors to SidCΔ100 occasionally produced a hybrid protein capable of translocating through the T4BSS. MavL was one such protein and, therefore, is considered a substrate of the T4BSS [4]. A common motif at the C-terminus of translocated effectors is a glutamate-rich stretch called the E-block [4]. These acidic residues were later found to promote the translocation of effectors through the T4BSS [5]. Inspection of the MavL C-terminus reveals a potential E-block motif consisting of three consecutive glutamate residues, which may be responsible for rescuing the translocation deficit present in SidCΔ100. 

MavL homologues are present in all sequenced *L. pneumophila* strains (15 genomes), with sequences being over 90% identical [6]. A homolog with 51% sequence identity was found in *L. quateirensis*, but no other *Legionella* species contain MavL homologs. Interestingly, many of the homologs start from residue corresponding to Met41 in MavL. This finding may point to an inaccurate start site annotation for MavL within the Uniprot database. Alternatively, MavL harbors an N-terminal extension, distinguishing it from some other homologues. Neither the structure nor the function of this effector has been elucidated. 

Secondary structure predictions using PsiPred [7] show MavL to be predominantly α-helical, with several short β-strands interspersed throughout. To gain potential insight into the function of MavL we determined its crystal structure and refined it to 2.65 Å resolution. The central segment of MavL has secondary structure arrangement similar to several classes of enzymes and is most reminiscent of ADP-ribose binding macrodomains, albeit with a different connectivity. Indeed, MavL binds ADP-ribose as detected by isothermal titration calorimetry (ITC). The structure reveals the presence of a pair of aspartate residues, where the catalytic glutamates are located in the poly-(ADP-ribose) glycohydrolases (PARGs). The presence of tandem acidic residues at this critical position may suggest hydrolase activity for MavL, although we were unable to demonstrate such an activity in vitro. These structural insights will be useful in guiding further biochemical investigations into the function of MavL. 

## 2. Materials and Methods

### 2.1. Bacterial Strains, Yeast Strains, and Growth Conditions

The bacterial strains, yeast strains, and plasmids used in this study are listed in Table 1. *Legionella* strains were grown in ACES [N-(2-acetamido)-2-aminoethanesulfonic acid]-buffered yeast extract (AYE) broth or on buffered charcoal yeast extract (BCYE) agar [8]. Antibiotics were added to the media when required; chloramphenicol, kanamycin, and/or isopropyl-β-D-thiogalactopyranoside (IPTG) were added to media at 6 µg/mL, 25 µg/mL, and 1 mM, respectively. *L. pneumophila* grown on solid media were incubated aerobically at 37 °C for 72 h, while broth cultures were grown overnight with shaking. *Escherichia coli* cultures were grown in Luria-Bertani (LB) broth or agar. When necessary, ampicillin, kanamycin, and/or chloramphenicol were added to LB broth or agar at 100 μg/mL, 100 μg/mL, and 12.5 μg/mL, respectively. *E. coli* grown on solid media or in broth were incubated aerobically at 37 °C overnight. Yeast strains were grown at 30 °C in yeast extract/peptone/dextrose (YPD) medium or yeast nitrogen minimal medium supplemented with 2% glucose and amino acids including histidine (20 mg mL^−1^), methionine (20 mg mL^−1^), tryptophan (20 mg mL^−1^), adenine (20 mg mL^−1^), uracil (20 mg mL^−1^), and leucine (30 mg mL^−1^), when necessary. 

### 2.2. Tissue Culture of Human-Derived Immortalized Cells

Human embryonic kidney 293T cells (HEK293T) expressing Fcγ-receptor (HEK-293 Fcrγ) and HeLa cells were maintained in Dulbecco’s Modified Eagle Medium (DMEM) with GlutaMAX-1 (Gibco, Waltham, MA, USA) and supplemented with 10% heat-inactivated Fetal Bovine Serum (Gibco). The human monocytic cell line, THP-1, was grown in RPMI 1640 medium GlutaMAX (Gibco^®^, Waltham, MA, USA). Heat inactivated HyClone^TM^ Foetal Bovine Serum (Thermo Fisher Scientific, Waltham, MA, USA) and penicillin/streptomycin were added at 10% and 1% of the concentration, respectively. All cell lines were grown in T75 cm^2^ flasks (Corning, Corning, NY, USA), incubated in 5% CO_2_ at 37 °C, and split at a cell-media ratio of 1:10 after 48–72 h growth.

### 2.3. Genetic Manipulation

The plasmids and primers used in this study are listed in Table 1 and Table 2, respectively. Cloning was carried out using primer-incorporated restriction sites (underlined sequences in Table 2) and genomic DNA of *L. pneumophila* strain 130b, or HeLa cell cDNA, as the template. TEM1-fusion constructs for translocation assays were made through the amplification of mavL (Lpw27521) (primers MavL_(pTEM1)F_ and MavL_(pTEM1)R_), before cloning corresponding product into pXDC61 harboring-TEM-1. Cell transfection constructs expressing GFP-tagged MavL and FLAG-tagged UBE2Q1 were generated through amplification of mavL (primers MavL_(pGFP)F_ and MavL_(pGFP)R_) and ube2q1 (primers UBE2Q1_(pFLAG)F_ and UBE2Q1_(pFLAG)R_), before cloning corresponding products into pEGFP-C2 and p3xFLAG-Myc-CMV™. To create plasmid p4HA-MavL expressing HA-tagged MavL during *Legionella* infections, mavL was amplified using primers MavL_(p4HA)F_ and MavL_(p4HA)R_, and the corresponding product was cloned into pMMB207c. To create plasmid pGBKT7-MavL for yeast two-hybrid screening, mavL was amplified using primers MavL_(pGBK)F_ and MavL_(pGBK)R_, and the corresponding product was cloned into pGBKT7. 

Plasmid constructs were maintained in *E. coli* through transformation of plasmid DNA into chemically competent XL1-Blue via heat-shock, in accordance with the protocols of the manufacturer. Plasmids were extracted using the AxyPrep™ plasmid DNA purification miniprep kit (Axygen, Corning, NY, USA). Transformation of plasmid DNA into *L. pneumophila* was performed via electroporation. Overnight cultures of *L. pneumophila* were grown in AYE broth, pelleted in PBS, washed with ice-cold water, and resuspended in water +10% (*v*/*v*) glycerol. Plasmid DNA was then added and *L. pneumophila* cells pulsed at 2.3 kV, before cells were grown in AYE broth and subsequently plated on BCYE with appropriate antibiotics for 48 h. Transformation of plasmid DNA into the yeast strain AH109 (MATa) was performed according to the lithium acetate method [14], and transformants were plated on selective media. 

### 2.4. TEM-1 Beta Lactamase Translocation Assay 

THP-1 cells were seeded at 8 × 10^4^ cells/well in 96-well tissue culture plates (Corning), phorbol-12 myristate 13-acetate (PMA) was added (10^−8^ M) to stimulate the differentiation of THP-1 monocytes into macrophages, and the cells were incubated for 24 h. *L. pneumophila* strains were grown in AYE broth with 1 mM IPTG and 12.5 µg/mL chloramphenicol. Cells were inoculated in triplicate with *L. pneumophila* strains (MOI of 1:40) for 1 h. Cells were then washed with Hanks’ Balanced Salt Solution (HBSS) supplemented with 5% (*v*/*v*) HEPES. Beta-lactamase-loading solutions (Life Technologies, Carlsbad, CA, USA) and CCF2-AM substrate (Invitrogen, Waltham, MA, USA) were added with 2.5 mM probenecid, in accordance with the protocols of the manufacturer. Cells were incubated at room temperature for 105 min before loading solutions were removed and replaced with HBSS + 5% HEPES + 2.5 mM probenicid. A ClarioSTAR microplate reader (BMG Labtech, Mornington, VIC, Australia) was then used to detect emissions at 450 nm and 520 nm after samples were excited with transmission of 410 nm light. The ratio of 450–520 nm light was calculated, and background fluorescence was subtracted and normalised against negative controls. An unpaired two-tailed Student’s t-test was applied to determine significance. At least three biological repeats of the translation assay were performed. 

### 2.5. Yeast Two-Hybrid Screening and Β-Galactosidase Assay 

The yeast two-hybrid (Y2H) screen was performed using *L. pneumophila* protein MavL as bait with the Matchmaker pre-transformed normalised HeLa cDNA library (Clontech, Mountain View, CA, USA), in accordance with the instructions of the manufacturer (Clontech PT3183-1). In this Y2H assay, the yeast strain AH109 was transformed with pGBKT7-*mavL* using the LiAc method and mated with the yeast strain Y187M pre-transformed with the pGADT7 RecAB plasmid carrying the HeLa cDNA library. Positive clones were selected by plating on quadruple dropout (QDO) media (Trp^−^, Leu^−^, Ade^−^, His^−^). Clones were also plated on low stringency or double dropout (DDO) SD media (SD/-Leu/-Trp), as a selection of only diploids with both prey and bait plasmids. Colonies were re-streaked on QDO, to ensure expression of reporter genes and, therefore, positive interaction. Colonies that were able to grow on both DDO and QDO plates were considered true positives, whereas colonies that could only grow on DDO plates were deemed false positives. The pGADT7-Rec-cDNA plasmids were isolated using the ZymoprepTM Yeast Plasmid Miniprep I Kit (Zymo Research Corp., Irvine, CA, USA), transformed into XL-1 Blue cells, and plated onto LB agar supplemented with ampicillin. The pGADT7-Rec-cDNA plasmids were then extracted and sequenced to identify the cDNA inserts.

Quantitative β-galactosidase assays were performed, in accordance with the protocols of the manufacturer (Clontech PT3024-1 manual), to measure the expression of the Y2HS *lacZ* reporter. Briefly, pairwise bait and prey plasmids (pGADT7, pGBKT7, pGBKT7-MavL, pGADT7-UBE2Q1) were transformed into *S. cerevisiae* strain AH109 using the LiAc method [14]. The yeast cells were selected for Trp^−^ and Leu^−^ plates and were grown overnight (OD600 of 0.6) before lysis in Z-buffer (60 mM Na_2_HPO_4_, 40 mM NaH_2_PO_4_, 10 mM KCl, 1 mM MgSO4, and 38.3 mM 2-mercaptoethanol, pH 7) and being assayed for the level of β-galactosidase activity using Ortho-nitrophenyl-p-D-galactopyraniside (ONPG) as a substrate. Data are from at least three biological repeats performed in triplicate. 

### 2.6. Co-Immunoprecipitation of EGFP and FLAG-Tagged Fusion Proteins 

HeLa cells were seeded in 10 cm tissue culture dishes. After 24 h, cells were transfected with 11 µg of purified p3xFLAG-Myc-CMV™-24 or pFLAG-UBE2Q1, plus pEGFP-Lem26 or pEGFP-MavL using FuGENE^®^ 6 (Promega, Madison, WI, USA), and incubated for 20 h. Cells were then washed 3 times with PBS before adding 600 µL KalB lysis buffer (50 mM Tris [pH 7.5], 150 mM NaCl, 1% *v/v* Triton X-100, 1 mM EDTA) supplemented with 1 mM Na3VO4, 1 mM NaF, 1 mM PMSF and Complete Protease Inhibitor (Roche, Burlington, MA, USA). Transfected cells were then pelleted and supernatants collected. Anti-FLAG M2 monoclonal magnetic beads (Sigma-Aldrich, Burlington, MA, USA) were added to the cell lysate supernatant, in accordance with the protocl of the manufacturer, and incubated on a rotating wheel at 4 °C for approximately 24 h. Cell lysate was removed and FLAG beads were washed 3 times with lysis buffer. FLAG peptide (Sigma-Aldrich, Burlington, MA, USA) was added to the beads at a concentration of 100 μg/mL and incubated for 30 min at 4 °C to elute FLAG-tagged proteins. Immunoprecipitation experiments were performed during at least three biological repeats.

### 2.7. Immunoblotting

Samples were prepared for SDS-PAGE gel electrophoresis, with the addition of lithium dodecyl sulphate (LDS) sample buffer and dithiothreitol (DTT) reducing agent, and heated to 70 °C for 10 min. Samples were then loaded onto NuPAGE™ 4–12% bis-tris gels (Life Technologies) and separated via electrophoresis at 165 V for 30 min in MES buffer (Life Technologies). Proteins were transferred to polyvinylidene difluoride (PVDF) membranes using the iBLOT-2™ transfer system (Life Technologies). After transfer, membranes were placed in a blocking solution of tris-buffered saline (TBS) with 5% (*w*/*v*) milk power and 0.1% Tween-20 (Chem Supply, Mitcham, VIC, Australia) for 1 h. After washing with TBS + 0.1% Tween-20, primary antibodies were applied overnight in TBS + 0.1% Tween-20 + 5% BSA at 4 °C. Primary antibodies used were: anti-FLAG-HRP conjugate (Sigma-Aldrich, Burlington, MA, USA) at 1:2000, anti-FLAG (Sigma-Aldrich) at 1:2000, anti-β-actin at 1:4000 (AC-15), anti-beta lactamase (QED Bioscience, San Diego, CA, USA) at 1:1500, and anti-GFP at 1:2000 (BioRad, Hercules, CA, USA). After further washing, secondary antibodies were then applied for 1 h. HRP-conjugated rabbit α-mouse and goat α-rabbit (Biorad, Hercules, CA, USA) antibodies were applied and were appropriate at 1:2000 dilutions in TBS + 0.1% Tween-20 + 5% BSA. Development reagents (GE Healthcare, Chicago, IL, USA) were applied to membranes, in accordance with the protocols of the manufacturer, and chemiluminescence was then visualised using a ChemiBIS imaging system (DNR-Bioimaging, Neve Yamin, Israel). 

### 2.8. Immunofluorescence Microscopy

To determine effector subcellular localization during *L. pneumophila* infection, HEK-293 Fcrγ cells were grown in 24-well tissue culture plates on coverslips and infected 16 h later with *L. pneumophila* 130b harboring p4HA vector or p4HA-MavL that had been cultured with 1 mM isopropyl β-D-1-thiogalactopyranoside (IPTG) to induce protein expression. Prior to infection, the *L. pneumophila* was diluted to a concentration of 10^8^ CFU/mL and coated with rabbit polyclonal anti-*L. pneumophila* (Meridian Life Science, Memphis, TN, USA) at 37 °C in 5% CO_2_ for 20 min prior to opsonization at an MOI of 1. Cells were infected at a multiplicity of infection of 10 for 16 h. Cells were then fixed in 4% paraformaldehyde-PBS for 20 min, then treated with 0.1% TritonX-100-PBS for 20 min and blocked with 3% BSA-PBS for 30 min. Cells were then incubated for 60 min in staining solution containing 0.2% BSA and a 1:50 dilution of anti-HA.11 monoclonal antibody (Covance, Burlington, NC, USA) for the detection of HA-fusions and a 1:50 dilution of anti-*Legionella* polyclonal antibody (Meridian Life Science), before the bound primary antibody was detected using a 1:1000 dilution of Alexa Fluor 488-conjugated anti-mouse antibody and Alexa Fluor 568-conjugated anti-rabbit antibody. For the last 10 min, HOECHST stain (1:20,000) was added to the staining solution. Coverslips were mounted onto glass slides with Dako Fluorescent Mounting Medium (Dako, Glostrup, Denmark). Immunofluorescence images were acquired using a Zeiss confocal laser-scanning microscope with a 100×/EC Epiplan-Apochromat oil-immersion objective. 

### 2.9. Cloning of Recombinant Mavl

The gene encoding MavL (*lpg2526*) was cloned into pMCSG7 and pRL652 vectors, by ligation independent cloning (LIC). Initially, a construct consisting of residues 2-444 was designed. This construct expressed and purified poorly, compelling us to explore alternative constructs. The PsiBLAST algorithm indicated a start site mis-annotation for MavL; most homologues had start sites corresponding to the MavL residue M41. Two constructs of MavL were designed to investigate this putative start site, namely MavL(42-435) and MavL(42-388). Inserts corresponding to these sequences were amplified from the MavL(2-444) plasmid and inserted into pMSCG7 and pRL652 by LIC.

### 2.10. Protein Expression and Purification

MavL(42-435) and MavL(42-388) in pMCSG7 were first transformed into BL21(DE3)pLysS and plated on LB agar containing ampicillin (100 µg/mL). A single transformant was inoculated into 20 mL of LB supplemented with ampicillin (100 µg/mL) and glucose (0.4%), and grown overnight at 37 °C. This culture was then sub-cultured into 1 L of terrific broth (TB) supplemented with ampicillin (100 µg/mL) and grown at 37 °C until an optical density (A600) of ~1.0 had been achieved, at which point the temperature was reduced to 18 °C and 500 µM of Isopropyl β-D thiogalactopyranoside (IPTG) was added to the culture to induce protein expression. The cells were then incubated for approximately 16 more hours before being pelleted at 6900× *g* for 15 min in a Beckman JLA 8.1000 rotor and stored at −80 °C. Cells were re-suspended in 30 mL of a lysis buffer (50 mM Tris, pH 8.0, 10% (*v*/*v*) glycerol, 0.1% (*v*/*v*) Triton X-100) and lysed two times at 35 kPsi in a cell disruptor (Constant Cell Disruption Systems, Kennesaw, Georgia). The lysate was spun at 21,000× *g* for 30 min in a Beckman JA25.50 rotor. Supernatant was added to 5 mL of Qiagen Ni-NTA beads pre-equilibrated with three column volumes of a buffer containing 20 mM Tris, pH 8.0, 50 mM NaCl and the beads were washed with 50 mL of this buffer. Protein was eluted with the same buffer supplemented with 100 mM imidazole. Purified protein was concentrated to 16 mg/mL in a 10 kDa molecular weight cut-off Millipore centrifugal filter span at 4000× *g*. Finally, the protein was loaded onto a Biorad SEC650 or S200 Increase (GE Healthcare) size exclusion column for separation of monomeric from dimeric species.

Limited proteolysis on MavL(42-435) and MavL(42-388) showed the latter construct to have greater susceptibility to degradation than the former. Furthermore, crystals were obtained only for a dimer species of MavL(42-435). This MavL(42-435) construct was, therefore, used in all downstream structural and ligand-binding studies.

Purification of MavL(42-435) on a Biorad SEC650 or GE S200 column allowed for the separation of dimer from monomer species, which were then concentrated to 15–20 mg/mL for crystallization trials. Initially, MavL(42-435) eluted from size exclusion predominantly in the monomeric form. The dimer species became more prominent after a day at 4 °C. By the third day, the dimer peak was larger than that of the monomer. At this point, further incubation of MavL(42-435) at 4 °C no longer increased the presence of the dimer. Re-running monomeric MavL on gel filtration regenerated some dimer and vice versa. These findings show that monomer and dimer species of MavL(42-435) are in equilibrium, with the monomer favored initially.

Intriguingly, only the MavL(42-435) dimer was found to crystallize. This observation comes in conflict with the previously reported equilibrium between monomer and dimer species. To account for this, it is possible that mixing purified MavL(42-435) with the crystallization solution stabilized the dimer. If so, rapid screening of the dimer after separation would have been essential to the crystallization of MavL(42-435). Consistent with this notion, screening of MavL was always carried out immediately after purification and any protein that could not be screened was flash-frozen in liquid nitrogen. In the few cases where protein was left at 4 °C, it gradually lost its crystallisability. 

No phasing model for MavL(42-435) was present in the protein data bank (PDB). We, therefore, opted to produce a seleno-methionine derivative for phasing by single anomalous dispersion (SAD). Inhibition of methionine biosynthesis was initially attempted in BL21(DE3)pLysS cells harboring the MavL(42-435) plasmid in pMCSG7. We found that growth of these cells in minimal media was prohibitively impaired. To overcome this, the MavL(42-435) plasmid was transformed into auxotrophic B834 *E. coli* competent cells. These transformants grew well in minimal media supplemented with methionine, but not without it. Induction of protein expression was carried out as described previously, using 500 µL IPTG and reducing the temperature to 18 °C. Importantly, the culture was resuspended in minimal media lacking methionine immediately prior to induction, at which point seleno-methionine was added to the culture. In this way, the incorporation of seleno-methionine into recombinant protein was ensured. Purification of seleno-methionine (SeMet) MavL(42-435) was carried out, as described for native MavL(42-435).

### 2.11. Crystallization of MavL(42-435)

Initial crystals for MavL(42-435) were obtained from Crystal Screen HT (Hampton Research, Aliso Viejo, CA, USA). Specifically, conditions A4 (0.1 M TRIS hydrochloride pH 8.5, 2.0 M Ammonium sulfate) and D3 (0.1 M HEPES sodium pH 7.5, 2% *v*/*v* Polyethylene glycol 400, 2.0 M Ammonium sulfate) produced poorly diffracting needle clusters in less than three days of growth. Extensive efforts to optimize the morphology of these crystals using additive- and grid-screens were met with little success. A few months later, an improved crystal form of MavL(42-435) was discovered in Crystal Screen condition B3 (0.2 M Ammonium sulfate, 0.1 M Sodium cacodylate trihydrate pH 6.5, 30% *w*/*v* Polyethylene glycol 8000). These slower growing crystals diffracted better than the initial needle-clusters. After optimization, the best crystals were obtained at 20 °C in drops containing 1 µL protein in 15 mM Tris-HCl, pH 8.0, 50 mM NaCl mixed with 1 µL reservoir comprised of 0.2 M Ammonium sulfate, 0.1 M Citrate pH 6.0, and 22% *w*/*v* Polyethylene glycol 8000. Both native and seleno-methionine derivative MavL produced optimal crystals in this condition.

### 2.12. Data Collection and Structure Determination

Seleno-methionine derivative MavL crystals were transferred into 1 µL mother liquor containing 25% (*v*/*v*) glycerol. Diffraction data was collected to 2.65 Å at the Canadian Macromolecular Crystallography Facility (CMCF) 08ID beamline, Canadian Light Source (CLS), using a Pilatus3 S 6M detector [15]. Integration and scaling were carried out using the XDS software package [16]. Initial electron density maps were obtained in Phenix using the hybrid substructure search to determine heavy atom (selenium) sites followed by phaser EP to calculate phase estimates for all remaining structure factors. The autobuild script was then used to arrive at a suitable model for further refinement [17]. Manual adjustments to this model were made in Coot [18], followed by computational refinement using phenix.refine [17]. Data collection and refinement detail are summarized in Table 3. The coordinates and structure factors have been deposited to the Protein Databank with ID 6OMI.

### 2.13. Isothermal Titration Calorimetry of MavL(42-435) with ADP-Ribose

Titrations were carried out using the Nano ITC instrument (TA Instruments, New Castle, DE, USA) and analyzed with the NANOANALYZE software (TA Instruments, New Castle, DE, USA. Briefly, 300 µM ADP-ribose was titrated into the calorimeter cell containing 30 µM HisMavL(42-435). Both ligand and protein were in a buffer containing 15 mM Tris-HCl, pH 8.0, and 50 mM NaCl. The experiment was carried out at 20 °C.

## 3. Results

### 3.1. Cellular Localization of MavL

**Figure 1:**
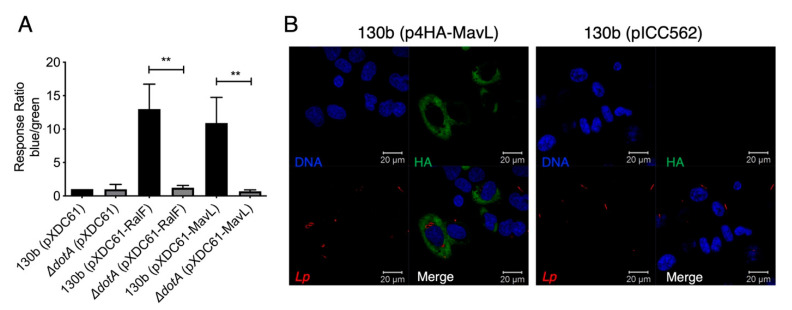
Translocation and localization of the Dot/Icm effector MavL during *L. pneumophila* infection. (**A**) TEM1-based translocation of *L. pneumophila* expressing a TEM1-MavL translational fusion compared to an isogenic Δ*dotA* derivative. Translocation is expressed as a response ratio of blue/green fluorescence, with background fluorescence subtracted and normalized against fluorescence seen in cells infected with *L. pneumophila* carrying the empty pXDC61 vector. TEM-RalF translocation was used as a positive control. Error bars represent the standard error of the mean (** denotes *p* < 0.01, unpaired two tailed *t*-test) of three biological repeats. (**B**). Immunofluorescence imaging of HEK-293 Fcrγ cells infected with *L. pneumophila* 130b (pHA-MavL) for 16 h; 4HA-MavL expression was induced with the addition of IPTG prior to infection (green); *L. pneumophila* 130b carrying pICC562 (empty vector) was used as a negative control for HA-staining. Bacteria were visualized with anti-*L. pneumophila* antibodies (red), DNA was visualized with Hoechst stain (blue).

A characteristic of *L. pneumophila* effector proteins is their translocation into the host cell via the Dot/Icm secretion system. Many Dot/Icm effectors then mimic host cellular machinery using eukaryotic-like domains [6,19]. To confirm the previous indirect report that MavL (Lpg2526/Lpw27521/Lpp2591) is translocated into host cells during *L. pneumophila* infection [4], a FRET-based TEM beta-lactamase translocation assay was performed according to the procedure described in [12]. MavL was cloned into pXDC61 to generate plasmid, pTEM1-MavL, encoding an N-terminal TEM1 beta-lactamase translational fusion with MavL. pTEM1-MavL was transformed into wild type *L. pneumophila* 130b; an isogenic Δ*dotA* mutant. *L. pneumophila* 130b carrying pTEM1-RalF was included as a positive control while Δ*dotA* pTEM1-RalF was included as negative a control (Table 1). THP-1 macrophages were infected with the described strains and fluorescence was measured at 450 and 520 nm. A media-only sample was included to allow for the subtraction of background fluorescence and finally the ratio of blue/green fluorescence was normalized to *L. pneumophila* 130b, carrying pXDC61 only. The results indicated that MavL was translocated into host cells in a Dot/Icm-dependent manner (Figure 1A). 

To examine the subcellular localization of MavL during *L. pneumophila* 130b infection, 4xHA-tagged MavL expression was induced in *L. pneumophila* by the addition of IPTG to cultures of *L. pneumophila* carrying the plasmid, p4HA-MavL (Table 1). *L. pneumophila*-carrying pICC562 was used as a control for anti-HA staining specificity. Bacteria were opsonized and used to infect HEK293T cells expressing Fcγ-receptor (HEK-293 FcRγ) for 16 h. Immunostaining with anti-HA antibodies showed that MavL staining occurred throughout the host cytoplasm but was excluded from the nucleus (Figure 1B).

### 3.2. MavL Associates with E2 Conjugating Enzyme, UBE2Q1

**Figure 2:**
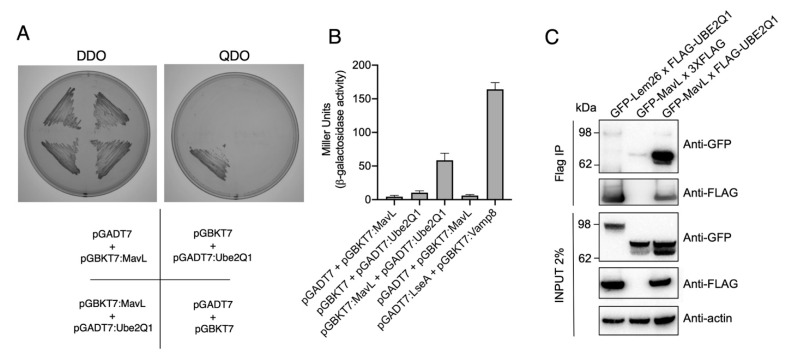
Protein-protein interaction between MavL and UBE2Q1. (**A**) Yeast two-hybrid analysis of protein-protein interactions in *S. cerevisiae* PJ69-4A. Growth on selective media for plasmid maintenance (DDO, double dropout) or selective media for interaction between proteins (QDO, quadruple dropout). Interactions on QDO shown for yeast strains carrying pGADT7:Ube2Q1 and pGBKT7:MavL. (**B**) β-galactosidase reporter activity for yeast strains shown in (**A**), and positive control carrying plasmids pGADT7:LseA and pGBKT7:Vamp8. LseA and Vamp8 are known interaction partners [13]. Activity is shown in Miller Units. (**C**) Immunoprecipitation of HEK293T cells transfected with pFLAG-UBE2Q1 and pGFP-MavL. Anti-FLAG immunoprecipitation was performed on cell lysates and eluates were subjected to immunoblot analysis with anti-GFP and anti-FLAG antibodies. The non-interacting partner GFP-Lem26 was included as a negative control. Actin, loading control.

To identify potential host cell binding partner(s) of MavL, we performed a yeast two-hybrid (Y2H) screen using a HeLa cDNA library as prey and full-length MavL as bait. Prior to screening the library, we confirmed that MavL did not auto-activate reporter gene expression. This was done by ensuring that *Saccharomyces cerevisiae* (Y2H Gold, Clontech) harbouring pGBKT7-*mavL* alone could not grow on quadruple knockout (QDO) selective yeast minimal media (YMM) (-Trp, -Leu, -His, -Ade). Screening for MavL eukaryotic interacting partners recovered approximately 500 positive prey clones. Sequencing of rescued cDNA plasmids from 50 randomly selected yeast colonies yielded only one MavL binding partner, the E2 conjugating enzyme, UBE2Q1 (nucleotides 1190–1360, encoding for amino acids 396-453). The interaction between MavL and UBE2Q1 was confirmed by re-transforming pGBKT7-*mavL* and pGADT7-*UBE2Q1* into *S. cerevisiae* strain AH109 and plating on selective media. *S. cerevisiae* growth was only observed on QDO YMM plates when both plasmids were present, supporting the interaction between MavL and UBE2Q1 (Figure 2A). Additionally, the relative affinity of MavL binding to UBE2Q1 was tested in a liquid β-galactosidase reporter assay. Yeast strains expressing both MavL and UBE2Q1 showed a more than five-fold increase in Miller units in β-galactosidase activity, compared with negative controls (Figure 2B). Yeast expressing empty vector and MavL, or UBE2Q1 alone, was used as a negative control. Yeast expressing the known interaction partners, Dot/Icm effector LseA and host VAMP8, was used as a positive control [13].

Next, we aimed to observe MavL interacting with UBE2Q1 in mammalian cells via co-immunoprecipitation (co-IP). HeLa cells were transfected with 3xFLAG-tagged UBE2Q1 and GFP-tagged MavL for 20 h, and co-IP was performed using whole cell lysates. The cell lysates were precipitated with anti-FLAG magnetic beads and analyzed by immunoblot using anti-FLAG and anti-GFP antibodies. GFP-MavL (70.7 kDa) was detected in the immunoprecipitate of cells co-transfected with 3xFLAG-UBE2Q1 (47.75 kDa), but not when co-transfected with 3xFLAG empty vector (0.8 kDa). In addition, an unrelated effector protein, GFP-Lem26 (112.19 kDa), was not detected after co-transfection and co-IP with 3xFLAG-UBE2Q1 (Figure 2C). This result supported a specific interaction between MavL and UBE2Q1. 

### 3.3. Crystal Structure of MavL

**Figure 3:**
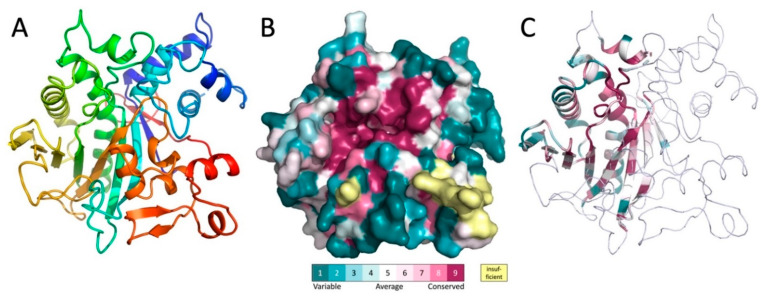
The structure of MavL and its similarity to other proteins. (**A**) The cartoon representation of MavL. The structure is rainbow colored from blue on the N-terminus to red on the C-terminus. (**B**) Surface representation of MavL color-coded by sequence conservation. The orientation is similar to that in panel A. Magenta indicates regions of high conservation. Of particular interest is the highly conserved deep depression toward the center of the protein. (**C**) MavL substructure that shows similarity to other proteins is shown in cartoon representation colored by sequence conservation, the remaining part of the structure is shown as thin ribbon. Orientation of the molecule is the same as in panel A.

To learn more about the biochemical function of MavL, the crystal structure of His-MavL(42-435) was solved by single anomalous dispersion (SAD) and refined to a resolution of 2.65 Å. This construct starts from the alternative start at Met41 and does not contain the 20 C-terminal residues, which are predicted disordered and contain the putative E-bloc translocation signal. There are three molecules in the asymmetric unit. They are nearly identical and superimpose with a root-mean-square deviation (rmsd) of ~0.4 Å. The entire molecule forms a single domain. The core of each molecule is formed by a highly curved, twisted, ten-stranded mixed β-sheet (Figure 3A). Two α-helices, one of them being 26-residues long with a kink in the middle introduced by the presence of a proline residue, line the concave side of the sheet and are nearly parallel to the β-strands. The convex side of the sheet is covered by several long loops with embedded short α-helices (Figure 3A). Among them, at the N-terminus of MavL, is a bundle of four short α-helices that are inserted between strands β1 and β2 and abate to one end of the β-sheet. 

The level of amino acid conservation along the MavL sequence was determined by sequence alignment with protein sequences from UniProt [20] and projected on the MavL structure using ConSurf webserver [21]. In addition to sidechains conservation in the interior of the protein that is likely necessary for the proper folding and protein stability, there is a large cluster of conserved residues surrounding a deep cleft in the MavL surface (Figure 3B), suggesting a functional role as a likely binding site for this cleft.

### 3.4. Structural Homologs of MavL

A quest for proteins with a similar fold to MavL was conducted using the DALI server [22,23]. Limited structural similarity was identified for MavL fragment encompassing ~100–130 residues to several other proteins, albeit with sequence identity below 10%. These structural features are located within amino acids 130–340, out of the 41-435 MavL sequence. The common structural features were limited to the several β-strands of the MavL central β-sheet and the two long α-helices on the concave side of the β-sheet (Figure 3C). Similar features are present in proteins containing the ADP-ribose (ADPR) binding macrodomain fold (e.g., PDB ID 3SIH/3SIG, [24]), β chain of tryptophan synthase (PDB ID 2O2E, 5TCG [25]), and PLP-dependent D-serine deaminase (PDB ID 3R0X, [26]), UDP-N-acetylmuramoyl:L-alanine ligase (MurC, PDB ID 2F00, [27]). This segment also displays structural similarity to the enzymes with the α/β-hydrolase fold [28], although the characteristic tight turn between the β-strand and following α-helix carrying the Ser nucleophile is absent. The superposition of MavL with each of these proteins superposes 50–75 Cα atoms with root mean squares deviation of ~1.5 Å. Importantly, all of the identified structural relatives are enzymes and, moreover, the substrates or cofactors associated with these enzymes are localized in the same region of the common structural framework, which coincides with the highly conserved surface of MavL. 

### 3.5. MavL Is Not Only Structurally Similar to the ADP-Ribose-Binding Macrodomains, but It Also Binds ADP-Ribose 

**Figure 4:**
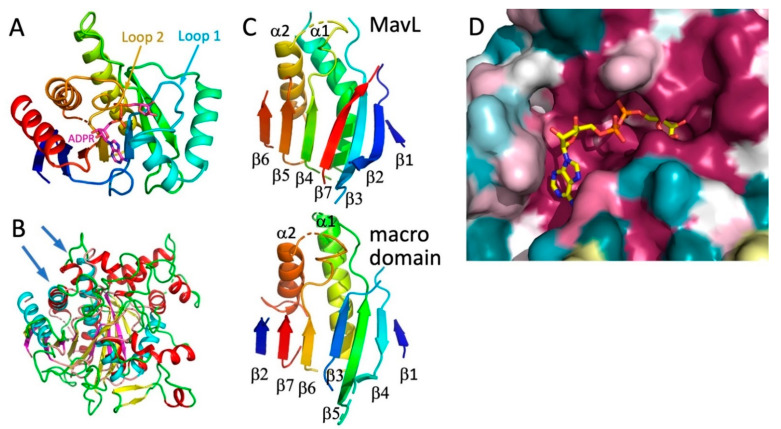
Comparison of MavL with macrodomains. (**A**) The compact macrodomain, PARP14 (ARTD8) macrodomain 1, with bound ADP-ribose (PDB ID 3Q6Z). This macrodomain has an additional N-terminal β-strand relative to the minimal fold. (**B**) Superposition of MavL and *T. curvata* macrodomain (PDB ID 3SIG), showing overlap of β-sheets and two long α-helices (marked by arrows). In MavL, β-strands are yellow, helices are red, and loops are green, while in the macrodomain, β-strands are magenta, helices are cyan, and loops are salmon. (**C**) While the secondary structural elements assume similar spatial positions in MavL and the *T. curvata* macrodomain, their connectivity is quite different. The secondary structures in both proteins are rainbow-coloured from blue at the N-terminus to red at the C-terminus. (**D**) The closeup of the cavity in the MavL structure as shown in panel 3B. The structure of *Tetrahymena thermophila* macrodomain (PDB ID 4EPP) was optimally superimposed on MavL, showing that the ADP-ribose bound to the macrodomain fits very snugly into a deep depression in the MavL surface formed by highly conserved residues.

A closer examination of the superpositions with the above-mentioned proteins suggests that the most likely functional homologs of MavL are the macrodomains. Therefore, next we performed a detailed comparison of MavL with various proteins containing macrodomains. The common core of ADPR-binding macrodomains contains ~200 residues and is an α/β/α sandwich with a curved central six-stranded mixed β-sheet covered on the convex side by three long α-helices and with two α-helices lining the concave face of the β-sheet. Most macrodomains contain additional α-helices and/or β-strands [29,30,31,32]. The ADPR binding pocket is located above the C-terminal ends of the central strands [24] (Figure 4A). The adenosine binding site is formed on top of two β-strands and an α-helix, while the pyrophosphate ribose binding site is formed predominantly by two loops named Loop 1 and Loop 2 [32] (Figure 4A).

The structurally most similar macrodomain to MavL, identified by DALI, was poly(ADP-ribose) glycohydrolase from *Thermomonospora curvata* (PDB ID 3SIG [24], which contains an additional 7-th β-strand relative to the minimal fold. Superposition of MavL and 3SIG structures show good correspondence for the seven β-strands, two of the three long α-helices on one side of the sheet, and one short α-helix on the other side (Figure 4B). However, while the spatial placement of β-strands is conserved, their connectivity/topology differs between MavL and the ADPR-binding macrodomain. In *T. curvata* macrodomain the order of strands is ↑β2-↑β7-↑β6-(α2)-↑β3-↑β5-(α1)-↓β4-↑β1. The order of the structurally corresponding strands in MavL is different, namely ↑β6-↑β5-↑β4-(α2)-↑β7-↑β3-(α1)- ↓β2-↑β1 (Figure 4C). The N- and C-terminal extension to the ADP-ribose binding macrodomain fold in MavL do not form separate domains but rather supplement the macrodomain fold, adding three strands to the central β-sheet and several α-helices (Figure 3A). Thus, despite the observed differences in β-strand connectivity between MavL and the macrodomain fold, the spatial arrangement of the key secondary structures in MavL results in a similar structural backbone that recreates the ADP-ribose binding present in macrodomains. Indeed, in the superposition of MavL with poly(ADP-ribose) glycohydrolase from *Tetrahymena thermophila* (PDB ID 4EPP [33], the ADP-ribose present in the latter structure fits very nicely into the deep groove on MavL surface (Figure 4D).

**Figure 5:**
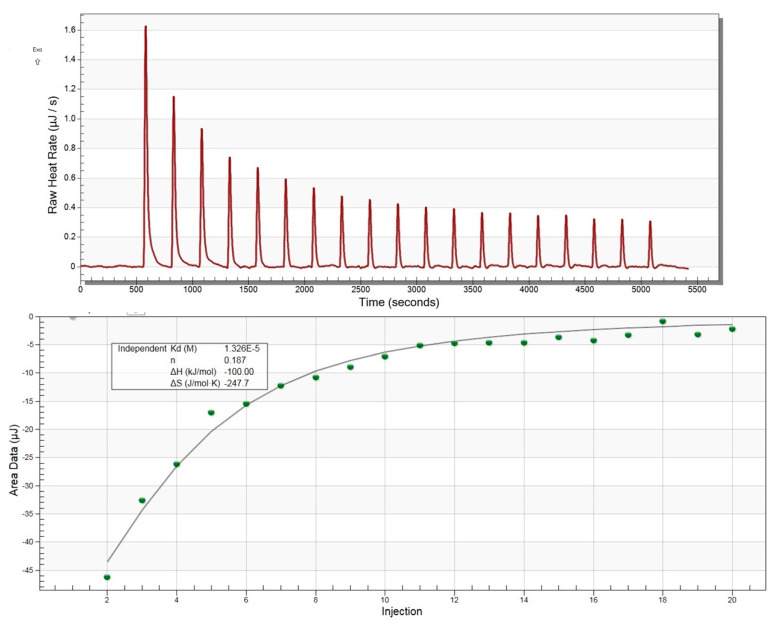
Isothermal titration calorimetry profile for ADP-ribose binding to MavL.

With these overall structural similarities between MavL and macrodomains in mind, as well as the presence of a deep cleft capable of accommodating ADPR, we next tested experimentally if MavL can bind ADP-ribose and, if so, what the binding constant would be. We have applied isothermal titration calorimetry (ITC) to test this hypothesis. The binding was easily detected by ITC and the binding constant Kd was estimated to be 13 µM (Figure 5), supporting the conclusions from the structural comparison.

### 3.6. MavL Is Most Similar to ADPR Glycohydrolases

ADP-ribose binding proteins can be classified as writers, readers, or erasers [34,35]. Writers are ADP-ribosyltransferases (ART) that synthesize ADP-ribose from NAD^+^ and modify target proteins on a specific amino acid. There are two classes of ARTs with similar fold, cholera toxin-like ARTCs, containing R-S-F-E motif and diphtheria toxin-like ARTDs containing H-Y-Y/F-E motif [36,37]. Reader domains recognize and bind ADP-ribose modifications and are usually embedded within large multidomain proteins. Finally, erasers bind mono-ADP-ribose (MAR) or poly(ADP-ribose) (PAR) covalently linked to a protein and hydrolyze the glycosidic bond to the protein or (1-2′) linkage to another ADPR, reversing the modification by writers [38]. We carried out a detailed analysis of the structural similarity between MavL and proteins, with macrodomains from each of these three classes, to identify which function is most likely for MavL.

MavL does not superimpose with the C-terminal catalytic writer domain of PARP14/ARTD8. This is not altogether surprising, as ARTD writers adopt a different overall fold than macrodomain readers. Briefly, writers contain an antiparallel twisted β-sheet sandwich flanked by several α-helices t [39,40]. MavL also lacks the H-Y-Y/F-E cluster within the putative ADPR binding site that is present in ARTDs [37]. It is, therefore, unlikely for MavL to display poly-ADP-ribose polymerase activity. 

Since MavL shares fold similarity with ADP-ribose-binding macrodomains, it is important to consider readers and erasers. Erasers differ from readers by a unique GGG-X_6-8_-QEE motif within their ligand-binding site that facilitates catalysis [34,38,41]. A mechanism for the glycohydrolase activity of *Thermomonospora curvata* PARG (PDB ID 3SIG) has been proposed, where the side-chain carboxyl of a glutamate from the conserved motif (Glu115) is deprotonated by the glycosidic oxygen that links ribose moieties in the chain [24]. This leads to the formation of a transient oxocarbenium intermediate that is neutralized by a water molecule. Prior to the reaction, a neighboring glutamate (Glu114) and phenylalanine (Phe227) residues orient the ribose moiety to facilitate cleavage [24]. Three glycine residues from the conserved motif interact with the pyrophosphate of a neighboring ADP-ribose moiety and are also essential for glycohydrolase activity in *T. curvata* PARG [24,41]. In contrast to *T. curvata* PARG, the reader macrodomains 2 and 3 of multidomain PARP14 (ARTD8, PDB ID 3VFQ) lack acidic residues corresponding to Glu114-Glu115 of PARG. Furthermore, Phe227 in PARG is replaced by a Leu1137 in PARP14, and no cluster of glycine residues is present in the corresponding position of PARP14, which results in a somewhat different orientation of the ribose moiety in these two binding sites. Thus, although ADP-ribose reader macrodomains and glycohydrolases share structurally similar ligand binding pockets, they can be distinguished by the presence/absence of the GGG-X_6-8_-QEE motif. 

**Figure 6:**
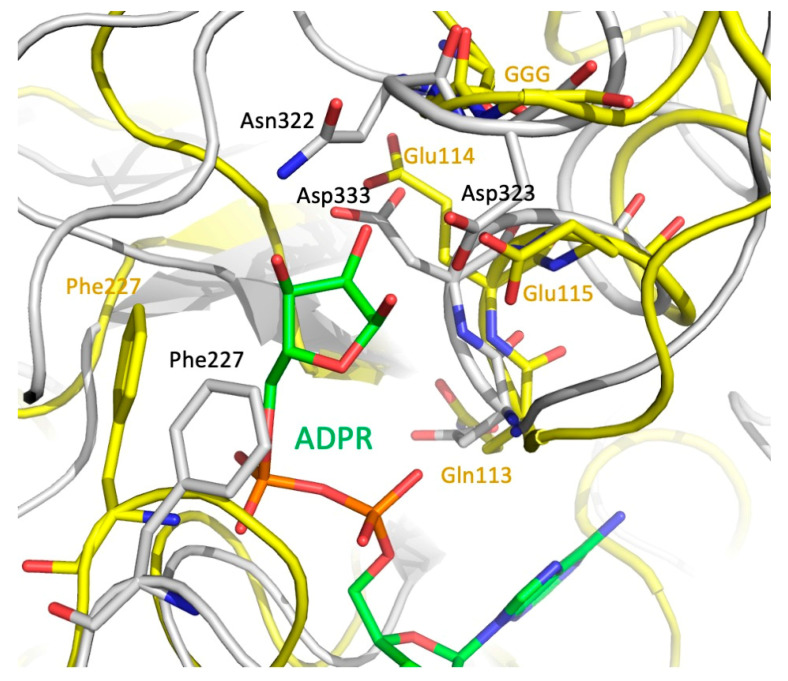
Optimal superposition of the eraser macrodomain from *T. curvata* (PDB ID 3SIG) (yellow backbone, green ADPR) and MavL (gray backbone). Closeup of the ribose-binding site. Sidechains of the GGG—QEE (Gln113Glu114Glu115) motif, the Phe sidechain in the eraser macrodomain, and the acidic residues and Phe in MavL are shown in stick mode. There are clear spatial similarities in positions of the key catalytic residues in macrodomain and the putative catalytic residues in MavL.

MavL does not contain the GGG-X_6-8_-QEE motif characteristic of eraser macrodomains, however, it contains an arrangement of acidic residues that are spatially equivalent to the acidic cluster in eraser macrodomains. Specifically, QE^114^E^115^ of *T. curvata* PARG (3SIG) superimposes with D^332^D^333^G in MavL, and the carboxylic groups of Asp333 and Glu114 are in close proximity. Moreover, the GGG of PARG are replaced by Gly321-Asn322-Asp323, with the acidic group of Asp323 assuming similar position to the catalytic Glu115 in PARG 3SIG (Figure 6). Additionally, Phe227 of PARG, which helps to correctly position the ribose of ADPR, corresponds spatially to Phe227 in MavL. Thus, structural analysis of MavL suggests that it has PARG activity and, by analogy to *T. curvata* PARG, Asp323 would initiate the cleavage reaction with Asp333 and Phe227 properly orienting the ribose moiety. Following this prediction, MavL was tested for ADPR glycohydrolases activity against one target, PARP1, which had been automodified with poly(ADP-ribose) (data not shown). However, in contrast to the human PARG control, MavL was unable to hydrolyze the ADP-ribose moieties attached to PARP1. This result indicates that MavL does not act as a PARG for poly-(ADP-ribosylated) PARP1. However, MavL could recognize a different ADP-ribosylated protein substrate, and further studies will be required to validate the glycohydrolase activity of MavL and to identify the host cellular target of this activity.

## 4. Discussion

The crystal structure of MavL(42-435) reveals partial fold similarity to several classes of enzymes that contain a mixed β-sheet flanked on one side by two long α-helices. The most likely similarity of functional relevance is to ADP-ribose-binding macrodomains. Interestingly, while the spatial localization of several central β-strands and two α-helices overlap, the connectivity of the secondary structure elements along the primary sequence in MavL differs from the classic macrodomains. Macrodomains function either as readers or erasers. The latter have a characteristic ADP-ribosyl glycohydrolase motif (GGG-X_6-8_-QEE) lining the ADP-ribose binding pocket. MavL lacks this specific motif, however, it contains several aspartates in the same part of the ADP-ribose binding site, with their acidic groups in similar positions to the two glutamates from the macrodomain eraser motif. The different connectivity of β-strands and placement of putative active site residues in MavL, while leading to the formation of a similar ADP-ribose binding site to macrodomains, strongly suggests a unique evolutionary history for MavL relative to the classical macrodomains, converging toward the same function. 

MAR- and PARylation is regulated by writers and erasers to mitigate the effect that would otherwise accompany unbridled modifications of this kind. Reversal of the PARP modification depends on enzymes with poly-(ADP-ribose) glycohydrolase (PARG) activity. Initial experiments to establish an ADP-ribosyl glycohydrolases activity using poly-(ADP-ribosylated) PARP1 as a substrate showed no detectable activity. Despite the similarity between MavL and eraser enzymes, these findings may point to a role for MavL as an ADP-ribose reader rather than eraser. If MavL does indeed have eraser activity, it could compensate for the effect of a bacterial or eukaryotic writer present in the host cell. 

Aberrant ADP-ribosylation can have severe consequences for the host cell, including the inhibition of protein synthesis or overproduction of cAMP. Many species of pathogenic bacteria have exploited this powerful modification using secreted effectors that exhibit mono- or poly-ADP-ribosyltransferase (MAR/PAR) activity. Indeed, the SidE family of Dot/Icm effectors rely extensively on ADP-ribosylation to ubiquitinate host proteins in an all-in-one approach. SidE effectors contain mono ADP-ribosyltransferase (mART) and phosphodiesterase (PDE) domains that act in concert to facilitate host protein ubiquitination. Initially, an ADP-ribose moiety is transferred to ubiquitin by the mART domain of SidE. Activity of the PDE domain then renders phosphoribosyl-ubiquitin, which is finally attached to a serine residue on the target protein. 

Interestingly, the *L. pneumophila* effector Lem26 (Lpg2523), encoded by a gene located in proximity to *mavL,* is a possible candidate for a writer as Lem26 contains a predicted phosphodiesterase (PDE) domain, which catalyzes the conjugation of ADP-ribosylated ubiquitin (ADPR-Ub) to a serine residue on substrates; Lem26 also causes a growth defect in yeast that is significantly reduced in the presence of MavL, suggesting a meta-effector relationship [42]. Moreover, since MavL and Lem26 do not interact directly [42], they may exhibit competing activities where MavL reverses the ADP-ribosylation caused by Lem26.

We found that MavL binds the E2 ubiquitin-conjugating enzyme, UBE2Q1, by yeast two-hybrid and co-immunoprecipitation. To rationalize why MavL binds UBE2Q1, it is convenient to imagine that Lem26 acts a writer and MavL acts as an eraser enzyme. If Lem26 or another host protein catalyzes the addition of ADP-ribose onto ubiquitin that is in complex with UBE2Q1, MavL could reverse this modification using eraser activity. In such a case, we should only expect MavL to bind ADP-ribosylated UBE2Q1 in a cellular context in vivo. It should be noted, however, that the function of Lem26 remains unknown and there is presently little published data on this protein. Still, the data presented here may point to meta-effector activity between MavL and Lem26, which warrants further exploration of the roles these proteins play in ADP-ribosylation and ubiquitination.

In summary, this study provides crystallographic and binding data that point to a putative role for MavL in ADP-ribosylation. Structural comparisons of MavL with ADP-ribose-binding macrodomains suggest reader or eraser activity against mono-ADP-ribosylated substrates. Although the biological role of MavL remains unclear, the studies presented here will hopefully guide further research toward this end.

## Figures and Tables

**Table 1:** Strains and plasmids used in this study.

Strains/Plasmids	Characteristics	Source
*L. pneumophila*		
130b (ATCC BAA-74)	O1; clinical isolate	[9]
∆*dotA*	130b ∆*dotA* in-frame markerless deletion	[10]
130b (pXDC61)	130b carrying pXDC61	[10]
130b (pTEM1-RalF)	130b carrying pTEM1-RalF	[10]
130b (pTEM1-MavL)	130b carrying pTEM1-MavL	This study
∆*dotA* (pTEM1-RalF)	∆*dotA* carrying pTEM1-RalF	[10]
∆*dotA* (pTEM1-MavL)	∆*dotA* carrying pTEM1-MavL	This study
130b (p4HA)	130b carrying p4HA	[11]
*∆dotA* (p4HA)	*∆dotA* carrying p4HA	[11]
130b (p4HA-MavL)	130b carrying p4HA-MavL	This study
*∆dotA* (p4HA-MavL)	*∆dotA* carrying p4HA-MavL	This study
		
*E. coli*		
XL-1 Blue	*recA1 endA1 gyrA96 thi-1 hsdR17 supE44 relA1 lac* [F*’ proAB lacI*^q^*ZΔM15* Tn*10* (Tet^R^)]	Stratagene
Plasmids		
pGEM^®^-T Easy	high copy cloning vector	Promega
pXDC61	vector expressing TEM1 version of BlaM,	[12]
pTEM1-RalF	pXDC61 expressing TEM1-RalF fusion protein	[13]
pTEM1-MavL	pXDC61 expressing TEM1-Lpw02526 fusion protein	This study
pICC562	pMMB207c based expression vector for IPTG inducible 4xHA-tagged proteins	[11]
p4HA-MavL	pICC562 expressing 4xHA-tagged MavL	This study
p3xFLAG-*Myc*-CMV™-24	Dual tagged N-terminal Met-3xFLAG and C-terminal *c-myc* expression vector	Sigma
pFLAG-UBE2Q1	vector expressing 3xFLAG-tagged UBE2Q1	This study
pEGFP-C2	vector to create C-terminal EGFP fusions	Clontech
pEGFP-MavL	vector expressing GFP-tagged MavL	This study
pEGFP-Lem26	vector expressing GFP-tagged Lem26	This study
pGADT7	GAL-4 activation domain expression vector	Clontech
pGBKT7	GAL-4 DNA binding domain vector	Clontech
pGBKT7-MavL	pGBKT7carrying *mavL*	This study
pGADT7-UBE2Q1	pGADT7 carrying UBE2Q1	This study
pGADT7-MavL	pGADT7 carrying *mavL*	This study
		
*Saccharomyces cerevisiae*		
Y187	*MATα* *ura3-52 his3-200 ade2-101 trp1-901 leu2-3,112 gal4Δ* *gal80Δ* *URA3::GAL1UAS-GAL1TATA-LacZ*	Clontech
AH109	*MATa, trp1-901, leu2-3, 112, ura3-52, his3-200, gal4* *Δ* *, gal80* *Δ* *, LYS2:GAL1UAS-GAL1TATA-HIS3, GAL2UAS-GAL2TATA-ADE2, URA3:MEL1UAS-MEL1TATA-lacZ*	Clontech
AH109 (pGADT7)	AH109 carrying pGADT7	This study
AH109 (pGBKT7)	AH109 carrying pGBKT7	This study
AH109 (pGBKT7 + pGADT7)	AH109 carrying pGBKT7 + pGADT7	This study
AH109 (pGBKT7-MavL)	AH109 carrying pGBKT7-*mavL*	This study
AH109 pGADT7-UBE2Q1_1190-1360_	AH109 carrying pGADT7-UBE2Q1	This study

**Table 2:** List of primers used in this study.

Oligo	Sequence (5′-3′)
MavL_(pTEM1)F_	CCCC GGA TCC ATG GCC TAT CAA TTA TTG CTC
MavL_(pTEM1)R_	CCCC AAG CTT TTA CTG AGG ACC CGA TTT TTT C
MavL_(p4HA)F_	CCCC GGA TCC ATG GCC TAT CAA TTA TTG CTC
MavL_(p4HA)R_	CCCC AAG CTT TTA CTG AGG ACC CGA TTT TTT C
MavL_(pGBK)F_	CCCCC GTCGAC CC ATG GCC TAT CAA TTA TTG CTC
MavL_(pGBK)R_	CCCC CTGCAG TTA CTG AGG ACC CGA TTT TTT C
UBE2Q1_(pGADT7)F_	CGC GAATTC ATGCAGCAGCCGCAGC
UBE2Q1_(pGADT7)R_	CGC GGA TCC GCCATCTTCTTTCGG
MavL_(pGFP)F_	CGCG GAA TCC ATG GCC TAT CAA TTA TTG CTC
MavL_(pGFP)R_	AGG ATC CCG TTA CTG AGG ACC CGA TTT TTT C
UBE2Q1_(pFLAG)F_	CCC GAATTC ATG CAG CAG CCG CAG CC
UBE2Q1_(pFLAG)R_	CCC GCGGCCGC TTA GCC GTC TTC TTT TGG GGG
Lem26_(pGFP)F_	CCCCGAGCTCGATGAAAACCAAACTAAACAAATC
Lem26_(pGFP)R_	CCCGTCGACTTAGTTTAGTGTAACCCACATA

**Table 3:** Data collection and refinement statistics.

Data Collection	
Space group	C121
Unit cell (Å)	97.6, 138.0, 108.5
Resolution (Å)	48.77–2.64 (2.66–2.64)
Total reflections	572,585 (85,399)
Unique reflections	82,310 (13,055)
Completeness (%)	99.30 (97.7)
Redundancy	6.96 (6.54)
Mean I/σ(I)	15.19 (1.37)
R_merge_ (%)	7.8 (138.7)
CC1/2	99.9 (64.9)
Refinement	
Resolution (Å)	45.14–2.64 (2.74–2.64)
No. of reflections	41,712 (4052)
R_work_/R_free_ (%)	21.7/25.5
No. of atoms/waters	8105/12
RMSD from ideal values	
Bond lengths (Å)	0.003
Bond angles (°)	0.62
Ramachandran plot (%)	
Favored	91.14
Allowed	8.02
Outlier	0.84

## Data Availability

The coordinates and structure factors have been deposited into the Protein Databank with ID 6OMI.
